# Molecular Modeling Study for Interaction between *Bacillus subtilis* Obg and Nucleotides

**DOI:** 10.1371/journal.pone.0012597

**Published:** 2010-09-07

**Authors:** Yuno Lee, Woo Young Bang, Songmi Kim, Prettina Lazar, Chul Wook Kim, Jeong Dong Bahk, Keun Woo Lee

**Affiliations:** 1 Division of Applied Life Science, Environmental Biotechnology National Core Research Center, Plant Molecular Biology and Biotechnology Research Center, Gyeongsang National University, Jinju, Gyeongsangnam-do, Republic of Korea; 2 Swine Science and Technology Center, Jinju National University, Jinju, Gyeongsangnam-do, Republic of Korea; University Paris 7, France

## Abstract

The bacterial Obg proteins (Spo0B-associated GTP-binding protein) belong to the subfamily of P-loop GTPase proteins that contain two equally and highly conserved domains, a C-terminal GTP binding domain and an N-terminal glycine-rich domain which is referred as the “Obg fold” and now it is considered as one of the new targets for antibacterial drug. When the Obg protein is associated with GTP, it becomes activated, because conformation of Obg fold changes due to the structural changes of GTPase switch elements in GTP binding site. In order to investigate the effects and structural changes in GTP bound to Obg and GTPase switch elements for activation, four different molecular dynamics (MD) simulations were performed with/without the three different nucleotides (GTP, GDP, and GDP + Pi) using the *Bacillus subtilis* Obg (BsObg) structure. The protein structures generated from the four different systems were compared using their representative structures. The pattern of *C_α_*-*C_α_* distance plot and angle between the two Obg fold domains of simulated apo form and each system (GTP, GDP, and GDP+Pi) were significantly different in the GTP-bound system from the others. The switch 2 element was significantly changed in GTP-bound system. Also root-mean-square fluctuation (RMSF) analysis revealed that the flexibility of the switch 2 element region was much higher than the others. This was caused by the characteristic binding mode of the nucleotides. When GTP was bound to Obg, its γ-phosphate oxygen was found to interact with the key residue (D212) of the switch 2 element, on the contrary there was no such interaction found in other systems. Based on the results, we were able to predict the possible binding conformation of the activated form of Obg with L13, which is essential for the assembly with ribosome.

## Introduction

GTP-binding proteins have been found in all living organisms and are involved in various essential cellular processes such as signal transduction, protein synthesis, membrane trafficking and, cell proliferation [1], [2]. These proteins belong to the GTPase superfamily whose sequence motifs are conserved in diverse species ranging from prokaryotes to eukaryotes [3]. Binding and hydrolysis of GTP effect the conformation of the GTP-binding proteins. The GTP and GDP-bound forms define the active and inactive states, respectively. Subfamilies of widely distributed bacterial GTP-binding proteins were discovered by the *Escherichia coli* (*E.coli*) Era and the *Bacillus subtilis* (*B.subtilis*) Obg (BsObg) proteins [1], [2], [4]. The Obg protein was originally identified in *B. subtilis* as a gene with GTP binding domain located downstream of *spoOB*. Consequently, Obg name originated from Spo0B-associated GTP-binding protein [5]-[7]. This protein was the first to be recognized as a member of a subfamily of GTP binding proteins that are conserved in both prokaryotic and eukaryotic cells [1], [2], [4], [8]. The Obg family GTPase implicated in the following diverse cellular processes including cell growth such as morphological differentiation and DNA replication [9]–[11], early steps of sporulation and for stress-dependent activation of the σ^B^ transcription factor that controls a cellular response to environmental stress [12], [13]. Moreover, ObgE, an Obg homolog in *Escherichia coli*, has also been reported to be involved in chromosome partitioning, the regulation of DNA replication as well as DNA repair process [14], [15]. Although the basic functions of the Obg subfamily proteins are not clearly established, the majority of bacterial Obgs have been commonly found to be associated with ribosome, in specific binding of Obg with ribosomal protein L13 was proved through an affinity blot assay method, implying that those proteins fundamentally could play a role in ribosome assembly or maturation [12], [16]–[22]. The Obg subfamily proteins such as Era, Obg, YjeQ, and YlqF which are also known as translation factor have been widely studied and hence they are also considered as a new target for antibacterial drug recently [23].

**Figure 1:**
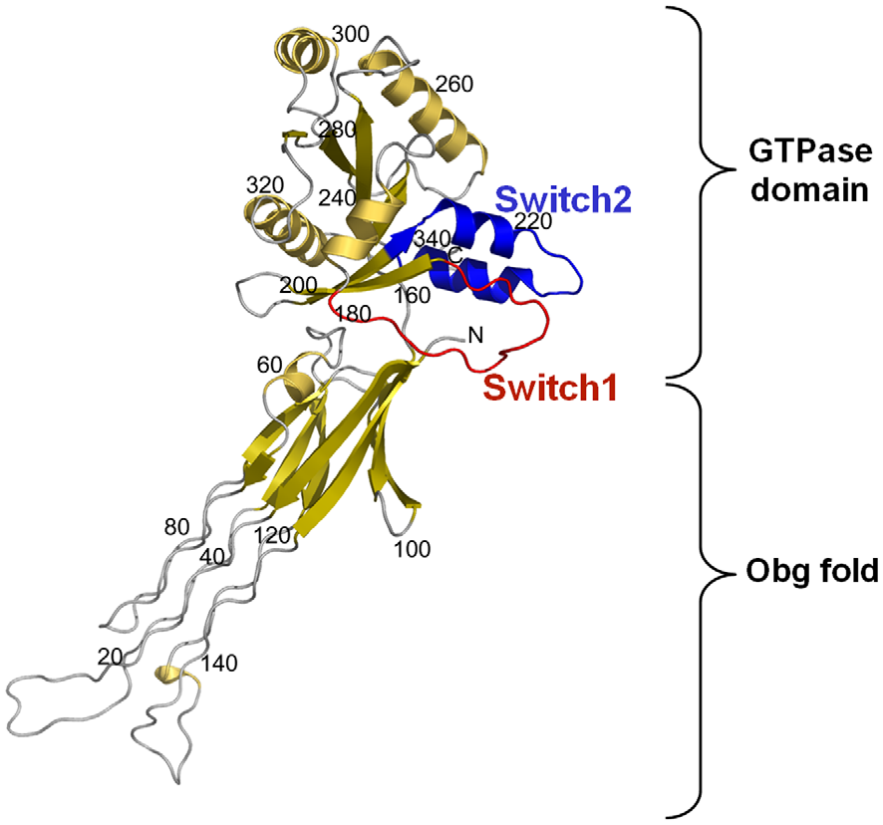
Key element of BsObg structure. Crystal structure of BsObg (PDB ID: 1LNZ) shows Obg fold (residues 1-158) and GTPase domain (residues 159–342) which comprises of switch 1 (red) and switch 2 (blue) elements. *C_α_* positions were numbered every 20 residues.

The crystal structures of both *B. subtilis* and *Thermus thermophilus* (*T. thermophilus*) Obg proteins were determined at 2.6 Å and 2.07 Å resolutions, respectively. The Obg protein contains three domains. The N-terminal glycine-rich domain (residues 1–158) which is referred as the “Obg fold” includes a sequence highly conserved among other members of the Obg family, but it doesn't identify any structural similarity with the other known proteins. The GTPase domain (residues 159–342) is a conserved GTP-binding domain similar to that found in small Ras-like GTPases [24], [25] (see Figure 1). The members of the Obg family share significant conservation along the switch 1 and switch 2 domains. These regions are also involved in protein-protein interactions [26]–[28]. The C-terminal domain (residues 343–428) which is called TGS domain is structurally similar to a domain found in bacterial stress response proteins. But TGS domain which was missing from BsObg structure is not widely conserved between Obg family members. The BsObg structure showed slightly different domain orientations between the molecules in the asymmetric unit [25], and the comparison between nucleotide-free *T. thermophilus* Obg and BsObg structures also revealed a dramatic domain rearrangement of Obg with significant conformational changes in the switch 1 and switch 2 regions [25]. However, no structural differences were observed between apo form and GDP-bound configurations of BsObg, implying that conformational changes associated with GDP binding are not sufficient to affect the Obg domain movement [24]. Altogether, these suggest that the orientation of N-terminal domain (Obg fold) of Obg proteins may be regulated by guanine nucleotides and further that the switch element recognition of GTP-bound configurations can trigger a conformational rearrangement between the domains [24], [25]. Thus, in order to fully understand the mechanism of the molecular switch of Obg protein, it is required to obtain the GTP-bound structure of Obg [24], [25], [29].

Here, we studied the structural change in switch element of BsObg due to GTP binding using molecular dynamics (MD) simulations. To investigate the effects and structural changes of GTP bound to Obg and GTPase switch elements for activation, four different molecular dynamics (MD) simulations were performed with/without the three different nucleotides (GTP, GDP, and GDP + Pi) using the currently available BsObg structure.

## Results

### 1. Stability of Obg protein structure during MD simulations

**Figure 2:**
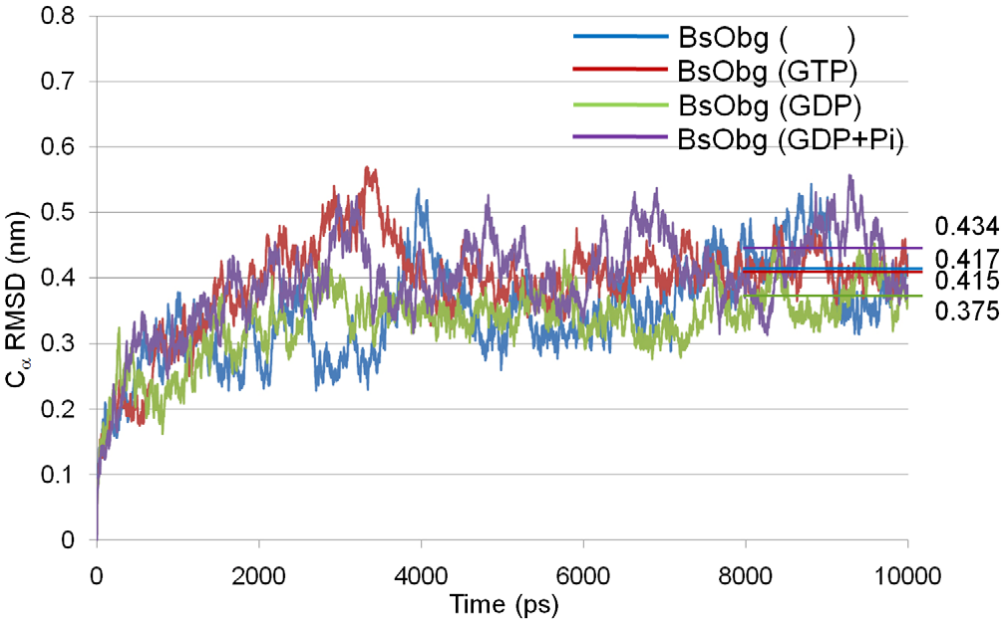
RMSD plots for four model structures during MD simulations. RMSDs of the *C_α_* atoms with respect to the starting coordinates over the four MD simulations were measured. Apo, GTP, GDP, and GDP+Pi-bound forms are in blue, red, green, and violet lines, respectively.

Four 10 ns MD simulations were successfully performed and analyzed. The details of the MD simulation environments and the size of the systems are listed in Table 1. In order to compare the protein structures from the four different systems, their representative structures were selected from each simulation and those are the most closed conformation to the average structure for the last 2 ns snapshots. The calculated average *C_α_* root-mean-square deviations (RMSDs) of each system (apo, GTP, GDP, and GDP + Pi) during the last 2 ns are 0.417, 0.415, 0.375, and 0.434 nm, respectively. The RMSD values of all the systems are slightly high because the structure has elongated N-terminal domain (Obg fold) (see Figure 2).

**Table 1:** Summary of four model systems for MD simulations.

No.	System	Nucleotides	No. of Atoms	Activity of protein
1	BsObg ( )	-	57,476	inactive
2	BsObg (GTP)	GTP	57,464	active
3	BsObg (GDP)	GDP	57,482	inactive
4	BsObg (GDP+Pi)	GDP+Pi	57,479	inactive

### 2. Comparison of entire structures (Obg fold, GTPase domain)

**Figure 3:**
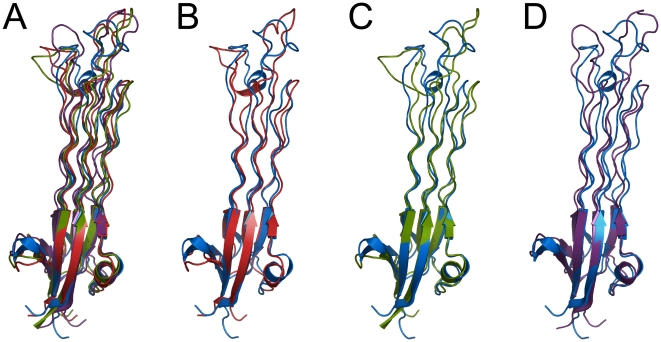
Structural comparison of Obg fold. Obg fold of each structure was superimposed for the comparison. The BsObg crystal structure in yellow was overlapped with the three model structures (A): apo (blue), GTP (red), GDP (green), and GDP+Pi-bound form (violet). And then the apo form was compared to the GTP (B), GDP (C), and GDP+Pi-bound form (D), respectively.

**Figure 4:**
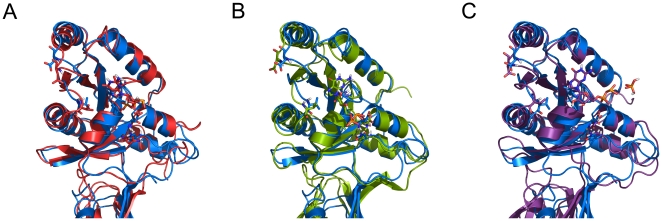
Structural comparison of GTPase domain. GTPase domain of each model structure was superimposed. Apo form (blue) was compared to the GTP in red (A), GDP in green (B), and GDP+Pi-bound form in violet (C), respectively.

**Figure 5:**
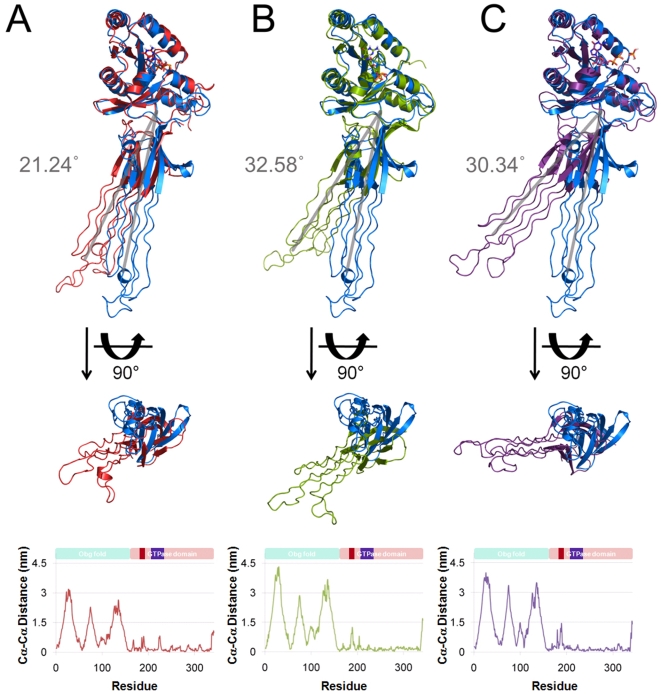
Structural comparison of the four model structures using MD simulation snapshots. Four model structures are aligned by superimposition of GTPase domain. Apo form (blue) was compared to the GTP in red (A), GDP in green (B), and GDP+Pi-bound form in violet (C), respectively. The bottom views for the aligned structures were visualized in the middle and finally *C_α_-C_α_* distances between the aligned structures are also provided.

During intra-domain comparison no significant structural differences were noticed in each Obg fold and GTPase domain (see Figure 3 and 4). However, we found a major change in the entire protein structure comparison. The angle between the Obg fold and GTPase domain in the GTP-bound system was significantly different from the others (see Figure 5). Hereafter, in our analysis apo form will be used as standard structure for the comparison with other systems. When the apo form was superimposed with other systems (GTP, GDP, and GDP+Pi) focusing on the Obg fold domain (residues 1–158), the RMSDs were 0.23, 0.29, and 0.29 nm, respectively (see Figure 3). Another superimposition using the GTPase domain (residues 159–342) resulted that the RMSDs were 0.26, 0.29, and 0.30 nm, respectively (see Figure 4). In order to study global motion of the protein for the systems, the bending angle of GTPase domain was compared. The angles between the apo form and each system (GTP, GDP, and GDP + Pi) were 21.24, 32.58, and 30.34°, respectively (see Figure 5). The GTP-bound form showed about 10 degrees difference compared to the other systems. The *C_α_-C_α_* distance plot also shows that the GTP-bound form only has different pattern from the rests. The average distances in the GTP-bound form were about 1 nm less than the rests (see Figure 5).

### 3. Differences of switch elements

**Figure 6:**
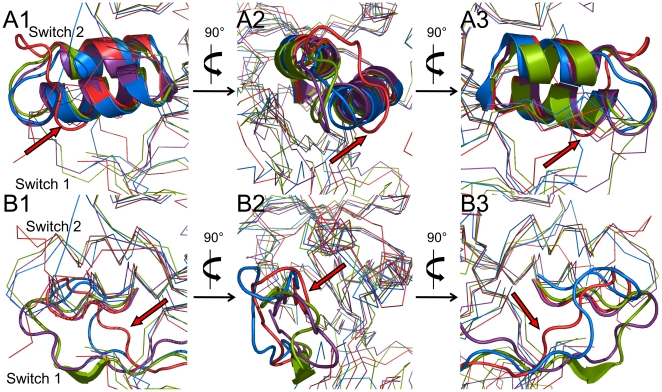
Structure comparisons for the two switch elements. The GTPase domain of apo form (blue), GTP (red), GDP (green), GDP+Pi-bound form (violet) were superimposed in order to observe structural changes in two key elements: switch1 (Lower deck) and switch2 (Upper deck). For clarity only the switch elements were represented as cartoons and the rest of the parts as lines. Each panel shows front view (A), left side view (B) and backside view (C). The arrows indicate the switch elements of the GTP-bound form.

The structures of switch 1 (residues 180–195) and switch 2 (residues 211–237) elements in nucleotide binding site were compared. In switch 2 element, we identified one substantial change in the loop region between the two helices of the GTP-bound system (see Figure 6A). The loop of the GTP-bound system was significantly shifted to the right site in the figure 6A1 compared to the rest. It can be regarded as the main position of the loop is different from the other systems. It is also noticed that the conformation of switch 1 element is severely changed in the GTP-bound system comparing with the GDP and GDP+Pi bound systems (see Figure 6B). The switch 1 element of the GTP-bound system was most shifted to the right site in the figure 6B1 and as a result the switch 1 element is the closest to the switch 2 element in the GTP-bound system (see Figure 6B2).

**Figure 7:**
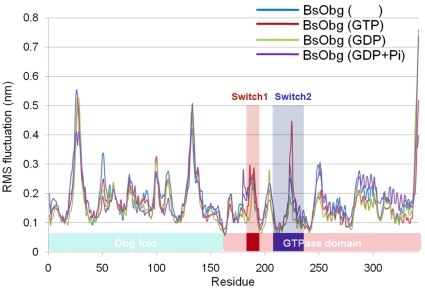
RMSF analysis of the two switch elements. RMSFs were monitored with respect to all the residues of the apo (blue), GTP (red), GDP (green), and GDP+Pi-bound form (violet), respectively.

RMSF analysis result showed that the flexibility of the switch 2 element in GTP-bound system was much higher than the other systems, while there is no remarkable difference in the switch 1 element (see Figure 7). Since the switch 1 element is located between the switch 2 element and the Obg fold, the switch 1 element seems to be used as the signal transporter between the two structures. Therefore the signal caused by the nucleotide substrates such as GDP or GTP will flow through the switch 2 element and then the switch 1 element and finally will reach to the Obg fold. From our analysis it is proposed that the flexible nature of the switch 2 element of the GTP-bound system is preferred to interact with and/or to control the switch 1 element and Obg fold, and eventually the function of the protein.

### 4. Essential dynamics (ED) analysis

**Figure 8:**
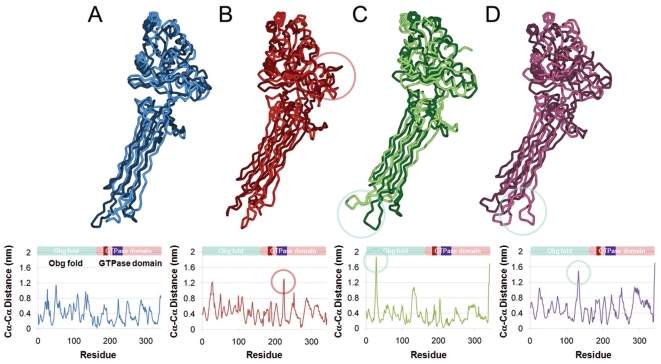
Essential dynamics analysis for the four model structures. The largest correlated motions were investigated for the Obg fold and switch elements of apo form (A, blue), GTP (B, red), GDP (C, green) and GDP+Pi-bound form (D, violet). For each panel, two protein trace structures from the maximal and minimal projections along the largest eigenvector were superimposed to show the largest correlated motions of each residue (minimum in light color and maximum in dark color). The *C_α_*-*C_α_* distance plots between the superimposed structures are also given to provide quantitative information.

The essential dynamics analysis was carried out to support our MD results and to understand the correlated motion of the switch elements in the four different simulations, and which identifies the essential movement in Obg during the simulation. We observed maximum movement of switch 2 element in the GTP-bound system compared to the other systems (see Figure 8B). A significant movement was also observed in the Obg fold of the GDP and GDP+Pi-bound systems (see Figure 8C and D) but no such motions were noticed in apo form (see Figure 8A). From these results, it can be concluded that each system has its own characteristics.

### 5. Binding mode of nucleotides

**Figure 9:**
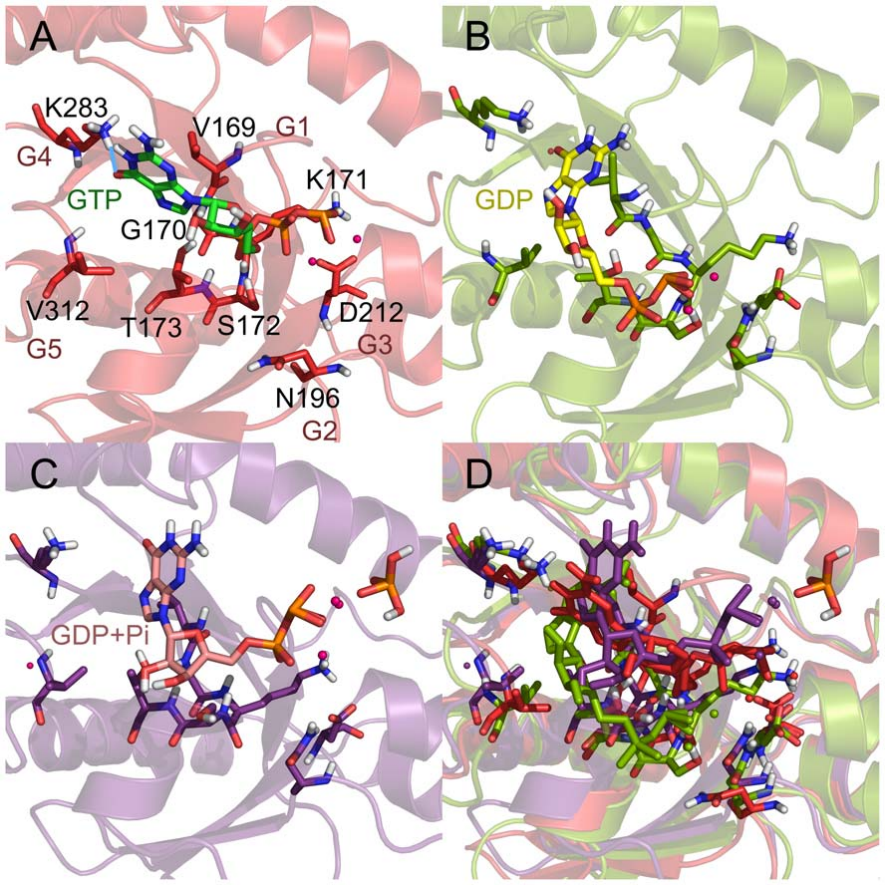
Binding mode of each nucleotide with the model structures. Binding conformations of each nucleotide in GTPase domains were compared along with residues having H-bond interaction: GTP (A), GDP (B), GDP+Pi (C), and all together (D). The nucleotides are shown in stick model.

**Figure 10:**
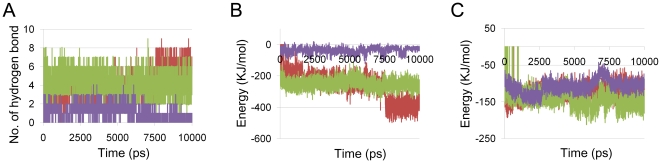
Interaction energy between the Obg protein and the nucleotides. The number of H-bonds (A), short range electrostatic energy (B) and short range Leonard-Jones potentials (C) of the GTPase domain with GTP (red), GDP (green) and GDP+Pi (violet) were monitored during the 10 ns MD simulation time.

When GTP was bound, its γ-phosphate oxygen was found to interact with the key residue (D212) of the switch 2 element, on the contrary to no interaction in other systems (see Figure 9). The GTP-bound system has seven H-bonding interactions compared to two for GDP and none for GDP+Pi-bound system. One hydrophobic interaction was observed in the GTP-bound system whereas other systems have three (see Figure 9 and Table 2). The oxygen of guanine ring has stable H-bonding interaction with K283 in GTP and GDP-bound systems whereas no such H-bonding interaction can be noticed in GDP+Pi-bound system. Based on this observation, it is concluded that the H-bond interaction plays main role in the recognition for the GTP. In order to understand the interaction energy between the protein and nucleotides, the number of intermolecular hydrogen bonds and the columbic energy were monitored and compared. It was shown that GTP is more stable with the Obg than others (see Figure 10).

**Table 2:** Key interactions between protein and nucleotides.

Nucleotide	Protein - nucleotides interactions	
	H-bonding	Hydrophobic
GTP	P_γ_-O…N K171 (0.294 nm)	V169
	P_α_-O_1_…O S172 (0.261 nm)	
	P_α_-O_1_…N^b^ S172 (0.262 nm)	
	P_α_-O_1_…N^b^ T173 (0.295 nm)	
	P_α_-O_2_…N T173 (0.276 nm)	
	P_α_-O_2_…N G170 (0.310 nm)	
	G-O…N K283 (0.269 nm)	
GDP	P_β_-O…N^b^ S172 (0.289 nm)	S168, V169, E250
	G-O…N K283 (0.290 nm)	
GDP+Pi	-	S168, T173, E250

N^b^ indicates backbone nitrogen. G-O indicates oxygen in guanine.

## Discussion

Upon binding of GTP, Obg protein becomes active. It is assumed that such a binding will lead to structural changes within the Obg fold, and subsequently, it triggers an important functional role. It is already suggested that the Obg fold may be the main platform for protein-protein interaction [24]. Our results also substantiate the importance of the Obg fold through its differential dynamic behavior in each system showing the possibility of Obg fold as the most favored part for protein-protein interaction. It was reported in the previous paper on the BsObg crystal structure that there is no conformational change between BsObg apo and GDP-bound form [24]. But according to our analysis of BsObg apo and GDP-bound form structures, we observed dramatic conformational changes in the Obg fold. In Figure 5, we showed the measured angle value of the Obg fold between simulated apo form and the representative structures of other nucleotide bound simulated systems. It clearly shows that apo form has more movement of the Obg fold during simulation, comparing to the other nucleotide bound systems, except GTP-bound Obg as a putative active form, which has unique structural changes.

In our protein-protein docking study based on the experimental evidences, we could be able to use the final putative activated form to investigate the specific binding mode between the Obg and ribosomal protein L13. The specific binding of Obg with L13 was already reported through an affinity blot assay method [12]. The interaction between the putative activated form of Obg and target protein (L13) was investigated through the protein-protein docking calculations. From this, two important H-bond interactions between Obg and L13 (Ser74 in Obg - Arg 144 in L13 and Arg24 in Obg - Val18 in L13) were predicted. Analyses of electrostatic potential surfaces of the Obg and L13 show that the Obg fold has highly conserved positively charged residues in the middle of the loop and this makes the loop highly electropositive in nature and gives way for the preferential interaction with L13, which is highly electronegative. This kind of binding and electrostatic information can be further used in structure-based drug design for finding a novel antibacterial drug [30].

In conclusion, four different MD simulations were successfully performed with/without the three different nucleotides (GTP, GDP, and GDP + Pi) to investigate the effects and structural changes of GTP bound to Obg and GTPase switch elements for activation. The protein structures from the four different systems were compared using their representative structures. Although no significant structural divergences were observed in each Obg fold and GTPase domain comparison, major structural changes were found in the relative orientation of the both domains. The angle between the Obg fold domains of simulated apo and GTP bound form was observed to be almost 10 degrees less than the angles of the other bound systems (GDP and GDP+Pi) with apo form. Also, the structures of switch 1 and switch 2 elements in nucleotide binding sites were compared. The flexible nature of switch 2 element probably leads to changes in switch 1 element conformation, which in turn may influence the Obg fold and its function. The switch 2 element was distinctively changed in the GTP-bound system due to the differential binding mode of the nucleotides. When GTP was bound to Obg, its γ-phosphate oxygen was found to interact with the key residue (D212) of switch 2 element, on the contrary no such interactions were observed in other systems. The number of intermolecular hydrogen bonds and the columbic energy were monitored and compared to understand the interaction between the protein and nucleotides. It demonstrated that the GTP is more stable with the Obg compared to other nucleotides. The conclusion and results of the present study were verified by the additional simulation studies (see Figure S1 and S2, Text S1).

Thus from the present study, we were able to find the 3D structure of the putative activated form of Obg and its structural properties and one possible reasons for its conformational changes. Also we were able to predict the possible binding conformation of the activated form of Obg with L13, which is essential for the assembly with ribosome.

## Methods

### 1. Starting structure preparation for MD simulation

(1) Protein structure

The 3D structure of BsObg (PDB ID: 1LNZ) was retrieved from the protein data bank (PDB). There are two monomers in apo and ppGpp-bound configurations. In previous study, no significant conformational differences between apo form and GDP or ppGpp-bound configurations were found [24]. The structure in apo configuration was taken for the present study. The hydrogen atoms and incomplete residues in the structure were added and fixed using *Discovery studio (DS)* (Discovery Studio 2.1, Accelrys Inc., San Diego, CA, USA).

(2) Nucleotide structures

The crystal structure of the Obg protein was complexed with ppGpp. We used the coordinates of ppGpp-bound form, and then, conserved binding mode of nucleotides. The four model systems were developed for MD simulations: apo form, GTP, GDP, GDP+Pi. Two magnesium ions (Mg^2+^) in the crystal structure were also considered for the simulation.

### 2. Computational details

The MD simulations were run on the Linux multi-node parallel cluster computer. All the MD simulations were carried out using the GROMACS program (version 3.3.1) [31], [32] with GROMOS87 force field. The Gromacs topology files for the nucleotides were generated using the PRODRG (http://davapc1.bioch.dundee.ac.uk/programs/prodrg/) [33]. The initial structure was immersed in an orthorhombic water box (0.8 nm thickness) and the net charge was neutralized by the addition of Cl^−^ and Mg^2+^ counterions. Long range electrostatics were handled using the particle mesh Ewald method [34]. In a system, protein alone consists of 3,336 atoms and the entire system is made up of approximately 57,500 atoms, which includes ∼18,300 water molecules (Table 1). The steepest descent energy minimization was used to remove possible bad contacts from the initial structures until energy convergence reached 2,000 kJ/(mol.nm). The systems were subject to equilibration at 300 K and normal pressure constant (1 bar) for 100 ps under the conditions of position restraints for heavy atoms and LINCS constraints [35] for all bonds. For all the systems considered for study, we performed 10 ns production run under periodic boundary conditions with NPT ensemble. Cutoff distances for the calculation of the electrostatic and Lennard-Jones interaction were 0.9 and 1.4 nm, respectively. The time step of the simulation was set to 2 fs, and the coordinates were saved for analysis every 1 ps.

### 3. Analysis

All visualizations were done using PyMOL and the *DS*. Trajectory analyses were carried out using tools built inside the GROMACS package. *C_α_* RMSDs and RMSFs were calculated by least squares fit. The protein structures from the four different systems were compared using their representative structure. The representative structures were selected from each simulation (last 2 ns) and those were the most closed conformation to the average structure. Essential Dynamics (ED) or principal component analyses were performed for the four different MD trajectories. The covariance matrices were diagonalized by making projections of trajectories on the eigenvectors. Movements of the 342 *C_α_* atoms of the protein in the essential subspace were projected along the most important eigenvectors from the analyses [36]. The number of H-bonds, short range (SR) electrostatic energy, and SR Leonard-Jones potentials of the GTPase domain with GTP were measured. For computing the number of hydrogen bonds, donor-acceptor distance cut-off value assigned was 0.35 nm.

## Supporting Information

Text S1 Supplementary results and discussion.
(0.03 MB DOC)

Figure S1 RMSD plot for eight additional model structures and binding mode of GTP structures with the model structures. (A) Root-mean-square deviations (RMSDs) of the *C_α_* atoms with respect to the starting coordinates over the eight additional MD simulations were measured. The RMSDs for 01_2702ps, 01_8806ps, 02_3330ps, 02_4365ps, 03_2099ps, 03_9581ps, 04_8265ps, and 04_9275ps systems are represented in light blue, dark blue, dark red, light red, light green, dark green, light violet, and dark violet lines, respectively. (B) Binding conformations of each GTP system (GTP-bound system in red, 02_3330ps in dark red, and 02_4365ps in light red) in GTPase domains were compared along with residues having H-bond and hydrophobic interaction. The GTP and interacting residues are shown in stick model.
(1.62 MB TIF)

Figure S2 Interaction energy of the nucleotides with the Obg protein in the six additional simulations. The number of H-bonds (A), short range electrostatic energy (B) of nucleotides with the GTPase domain in additional systems were monitored during the 2 ns MD simulation time.
(0.76 MB TIF)
