# Supplementary material for: Molecular Modeling Study for Interaction between Bacillus subtilis Obg and Nucleotides
Source: PLoS One. 2010 Sep 7;5(9):e12597. doi: 10.1371/journal.pone.0012597 (PMC2935376; doi:10.1371/journal.pone.0012597)
Supplement: Text S1 — Supplementary results and discussion. (0.03 MB DOC) [file pone.0012597.s001.doc]

**Molecular Modeling Study for Interaction between
*Bacillus subtilis* Obg and Nucleotides**

**Supplementary information**

Yuno Lee^1^, Woo Young Bang^1,2^, Songmi Kim^1^, Prettina Lazar^1^,
Chul Wook Kim^2^, Jeong Dong Bahk^1^ and Keun Woo Lee^1^*

^1^Division of Applied Life Science (BK21 Program),
Environmental Biotechnology National Core Research Center (EB-NCRC),
Plant Molecular Biology and Biotechnology Research Center (PMBBRC),
Gyeongsang National University (GNU), 900 Gazwa-dong, Jinju 660-701, Republic of Korea.

^2^Swine Science and Technology Center, Jinju National University, Jinju 660-758, Republic of Korea.

*Email: [kwlee@gnu.ac.kr](mailto:kwlee@gnu.ac.kr)

**Supporting results and discussions**

**Consensus verification for nucleotide binding conformations**

In order to verify whether the nucleotide bound conformations obtained from 10 ns MD simulations reappear or not, additional eight 2 ns MD simulations (01_2702ps, 01_8806ps, 02_3330ps, 02_4365ps, 03_2099ps, 03_9581ps, 04_8265ps, and 04_9275ps) were performed with different initial conformations which were chosen based on its RMSD calculated using 0 ps conformation as reference. The highest and lowest RMSD snapshots for each system were selected from each of the 10 ns trajectory in simulation time from 2 to 10 ns (see Figure 2). For example, the starting conformation of the 01_2702ps system is chosen from the 10 ns MD simulation of the apo system at 2702 ps (the lowest RMSD), 01_8806ps from 8806 ps (the highest RMSD). The 01 was denoted for apo system, 02 for GTP-bound system, 03 for GDP-bound system, and 04 for GDP+Pi-bound system. The 02 systems only reversely have the highest RMSD value at 3330ps rather than later time. Figure S1A shows the RMSD of the *C_α_* atoms with respect to the each of the 2 ns trajectories. The RMSD values of all the 2 ns systems are fluctuated like that of the 10 ns systems. The final snapshots of the eight systems are selected as representative structure.

When the GTP-bound system was superimposed with other additional systems (02_3330ps and 02_4365ps) focusing on the GTPase domain, the RMSDs were 0.20 and 0.15 nm, respectively. Comparison of GTP binding modes showed that the most of interacting residues (K171, S172, T173, and K283) in the additional systems (02_3330ps and 02_4365ps) are conserved with those of 10 ns GTP-bound system which are forming Hydrogen bonds and having a similar binding pattern (see Figure S1B). Similar interactions between the protein and nucleotides which are showing better stability of the GTP within the Obg compared to other nucleotides were observed from comparisons of number of hydrogen bonds and columbic energy for all additional systems (see Figure S2).

These results suggested that the computational approaches and the simulated structures were reliable.

**Legends of supplementary figures**

**Figure S1. RMSD plot for eight additional model structures and binding mode of GTP structures with the model structures.** (A) Root-mean-square deviations (RMSDs) of the *C_α_* atoms with respect to the starting coordinates over the eight additional MD simulations were measured. The RMSDs for 01_2702ps, 01_8806ps, 02_3330ps, 02_4365ps, 03_2099ps, 03_9581ps, 04_8265ps, and 04_9275ps systems are represented in light blue, dark blue, dark red, light red, light green, dark green, light violet, and dark violet lines, respectively. (B) Binding conformations of each GTP system (GTP-bound system in red, 02_3330ps in dark red, and 02_4365ps in light red color) in GTPase domains were compared along with residues having H-bond and hydrophobic interaction. The GTP and interacting residues are shown in stick model.

**Figure S2. Interaction energy of the nucleotides with the Obg protein in the six additional simulations.** The number of H-bonds (A), short range electrostatic energy (B) of nucleotides with the GTPase domain in additional systems were monitored during the 2 ns MD simulation time.
