# Supplementary figures and images for: Molecular Modeling Study for Interaction between Bacillus subtilis Obg and Nucleotides
Source: PLoS One. 2010 Sep 7;5(9):e12597. doi: 10.1371/journal.pone.0012597 (PMC2935376; doi:10.1371/journal.pone.0012597)

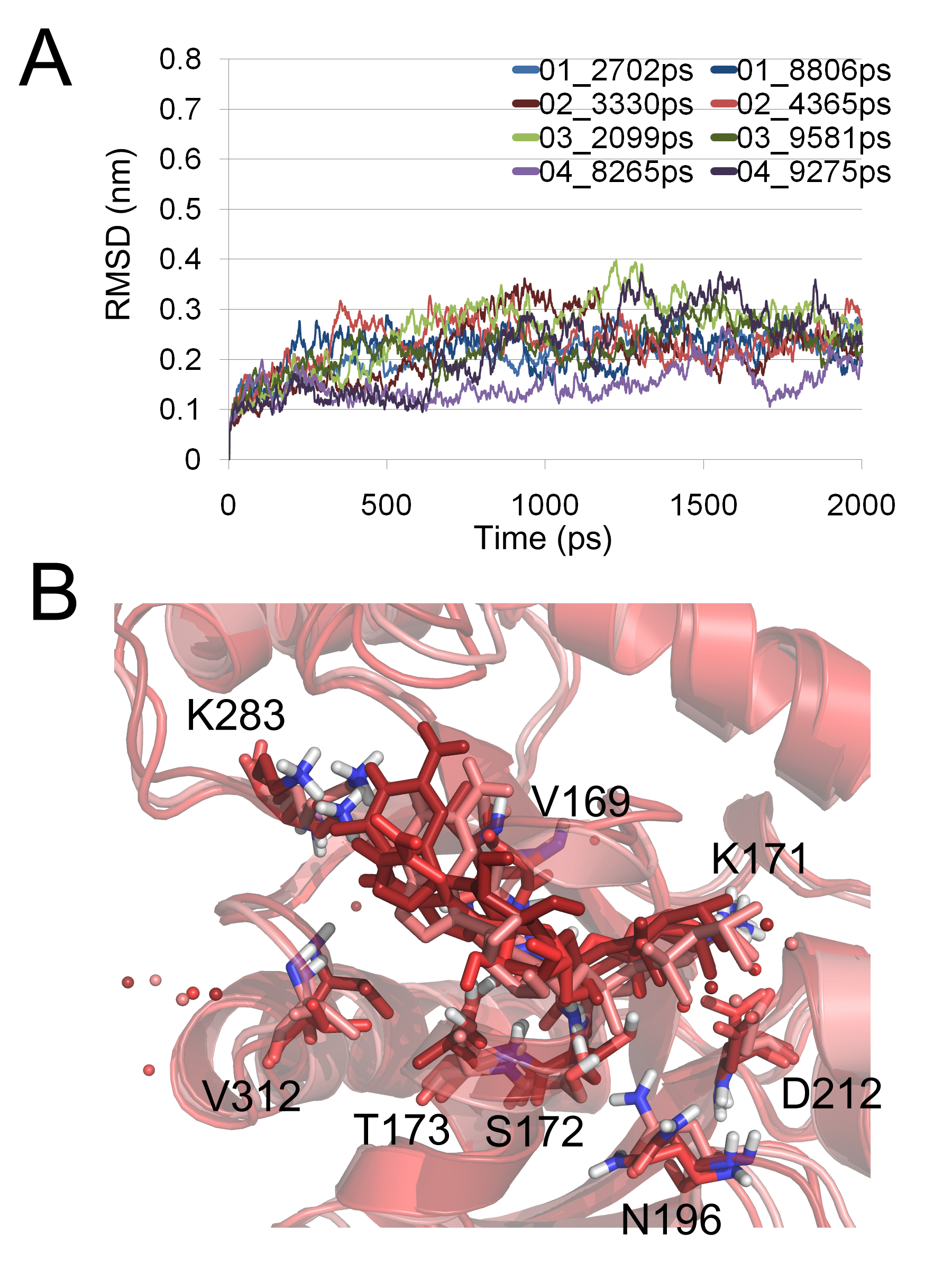

Supplement: Figure S1 — RMSD plot for eight additional model structures and binding mode of GTP structures with the model structures. (A) Root-mean-square deviations (RMSDs) of the Cα atoms with respect to the starting coordinates over the eight additional MD simulations were measured. The RMSDs for 01_2702ps, 01_8806ps, 02_3330ps, 02_4365ps, 03_2099ps, 03_9581ps, 04_8265ps, and 04_9275ps systems are represented in light blue, dark blue, dark red, light red, light green, dark green, light violet, and dark violet lines, respectively. (B) Binding conformations of each GTP system (GTP-bound system in red, 02_3330ps in dark red, and 02_4365ps in light red) in GTPase domains were compared along with residues having H-bond and hydrophobic interaction. The GTP and interacting residues are shown in stick model. (1.62 MB TIF) [file pone.0012597.s002.tif]

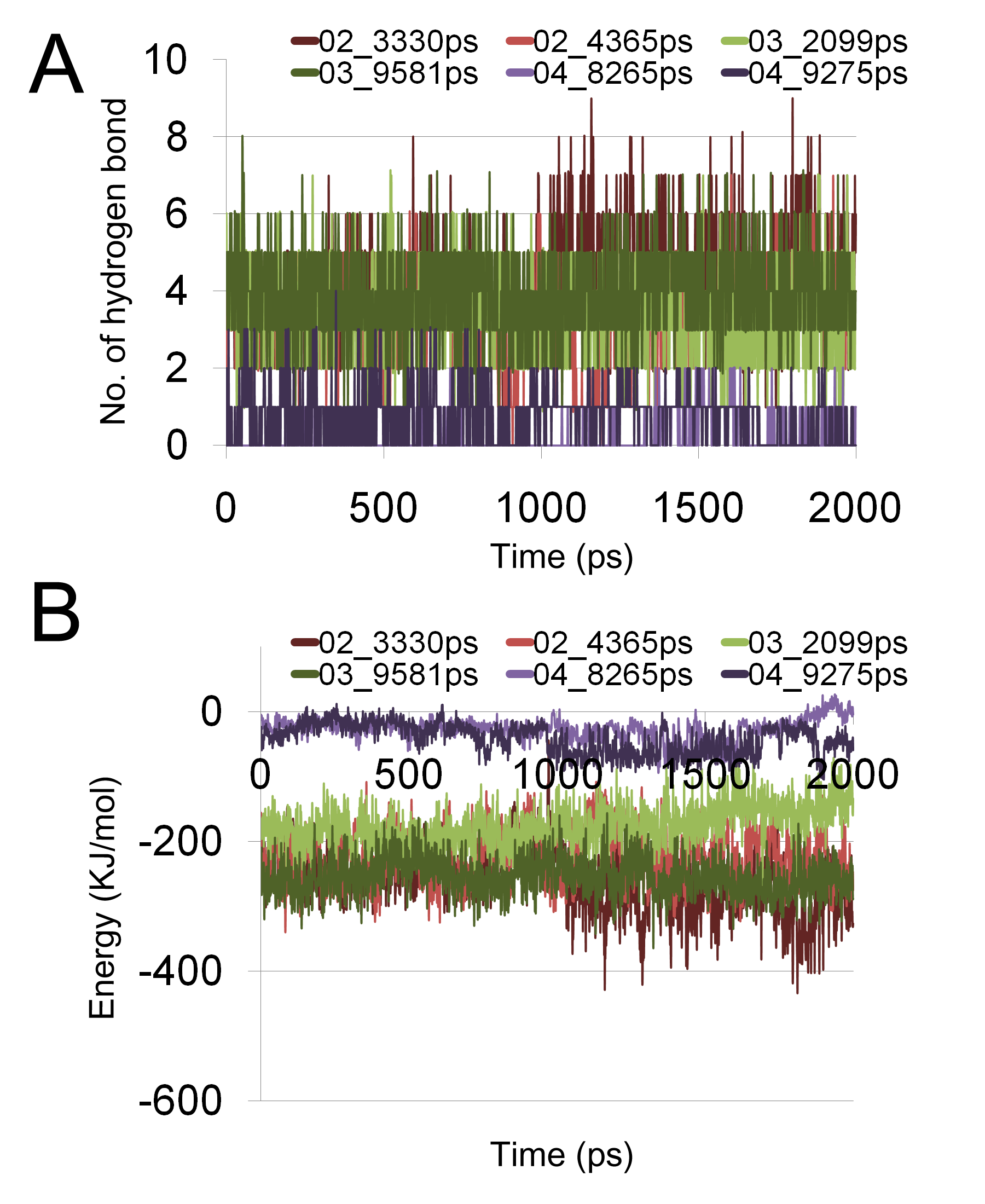

Supplement: Figure S2 — Interaction energy of the nucleotides with the Obg protein in the six additional simulations. The number of H-bonds (A), short range electrostatic energy (B) of nucleotides with the GTPase domain in additional systems were monitored during the 2 ns MD simulation time. (0.76 MB TIF) [file pone.0012597.s003.tif]
